# NK cell exhaustion in the tumor microenvironment

**DOI:** 10.3389/fimmu.2023.1303605

**Published:** 2023-11-02

**Authors:** Hao Jia, Hongmei Yang, Huaxing Xiong, Kathy Qian Luo

**Affiliations:** ^1^ Faculty of Health Sciences, University of Macau, Taipa, Macao SAR, China; ^2^ Ministry of Education Frontiers Science Center for Precision Oncology, University of Macau, Taipa, Macao SAR, China

**Keywords:** cancer, tumor microenvironment, NK cell, exhaustion, immunotherapy

## Abstract

Natural killer (NK) cells kill mutant cells through death receptors and cytotoxic granules, playing an essential role in controlling cancer progression. However, in the tumor microenvironment (TME), NK cells frequently exhibit an exhausted status, which impairs their immunosurveillance function and contributes to tumor immune evasion. Emerging studies are ongoing to reveal the properties and mechanisms of NK cell exhaustion in the TME. In this review, we will briefly introduce the maturation, localization, homeostasis, and cytotoxicity of NK cells. We will then summarize the current understanding of the main mechanisms underlying NK cell exhaustion in the TME in four aspects: dysregulation of inhibitory and activating signaling, tumor cell-derived factors, immunosuppressive cells, and metabolism and exhaustion. We will also discuss the therapeutic approaches currently being developed to reverse NK cell exhaustion and enhance NK cell cytotoxicity in the TME.

## Introduction

Natural killer (NK) cells are an important component of the innate immune system and play a critical role in controlling malignancies and viral infections (1). Unlike T cells, NK cells kill tumor and virus-infected cells without antigen presentation, allowing NK cells to elicit a rapid immune response (2). NK cell-based therapies are currently being developed for the treatment of blood cancers as well as various solid tumors (3, 4). However, in the tumor microenvironment (TME), NK cells always exhibit an exhausted state, which facilitates the immune escape of tumor cells and reduces the efficacy of NK cell-based therapies (5). The main features of NK cell exhaustion include impaired cytotoxicity, decreased secretion of cytokines, upregulated expression of inhibitory receptors, downregulated expression of activating receptors, dysregulation of proliferation, and metabolic dysfunction (6, 7). In recent years, the mechanisms of NK cell exhaustion have been intensively studied, although the exact mechanisms have not been fully elucidated. In this review, we summarize the current understanding of the mechanisms of NK cell exhaustion in the TME.

## NK cell maturation, localization, homeostasis, and cytotoxicity

NK cells arise from hematopoietic stem cells in the bone marrow. Mouse NK cells develop mainly in the bone marrow, whereas human NK cells can develop in the bone marrow as well as in other lymphoid tissues such as the thymus, spleen, lymph nodes, and liver (8, 9). Mouse NK cells are defined as CD3^−^ NK1.1^+^ cells and can be classified into four types based on the presence of two markers, CD27 and CD11b, to indicate their maturation stages. CD27^−^ CD11b^−^ NK cells are immature NK cells with high differentiation potential. These NK cells differentiate into CD27^+^ CD11b^−^ NK cells and then further develop into CD27^+^ CD11b^+^ NK cells that can migrate from bone marrow to peripheral tissues. CD27^−^ CD11b^+^ NK cells are the mature NK cells with high cytotoxicity (10–15). Human NK cells are defined as CD3^–^ CD56^+^ cells and can be further divided into two types: CD56^bright^ CD16^–^ and CD56^dim^ CD16^+^ cells. CD56^bright^ CD16^–^ NK cells account for only 5-10% of NK cells and are less mature and less cytotoxic. They are thought to be the precursors of CD56^dim^ CD16^+^ NK cells, i.e., mature NK cells. CD56^dim^ CD16^+^ NK cells account for 90% of NK cells and have higher cytotoxicity (10, 12, 14).

NK cells are widely distributed in various tissues and organs of mammals. In recent decades, studies have shown that NK cells are mainly found in bone marrow, peripheral blood, liver, lung, spleen, and uterus. In addition, there are also significant numbers of NK cells in lymph nodes, thymus, mucosa-associated lymphoid tissues, skin, and kidney (16–20). In peripheral blood, 5-20% of lymphocytes are NK cells (9). In the liver, NK cells are enriched in the hepatic sinusoids. In mice, NK cells account for 5-10% of liver lymphocytes, whereas this proportion can reach 30-50% in humans (16). In the lung, NK cells can account for approximately 10-20% of total lymphocytes (21–23). Although NK cells account for only 2-4% of splenic lymphocytes in mice, their absolute number is quite high (21, 24). NK cells *in utero* are specialized NK cells that can make up 70% of lymphocytes at the maternal-fetal interface (25, 26).

Adequate numbers of NK cells in peripheral tissues are important for normal immune surveillance function. NK cells are continuously produced by development and differentiation from hematopoietic stem cells in the bone marrow and are replenished to peripheral tissues to replace dead NK cells (27, 28). Previously, mature NK cells were thought to have low proliferation ability. However, recent studies have shown that mature NK cells, similar to T cells, can undergo homeostatic proliferation to contribute to maintaining their numbers in peripheral tissues. This homeostatic proliferation depends on stimulation by several cytokines, including IL-2 and IL-15 (28–30). NK cells even have some memory properties similar to those of long-lived CD8^+^ T cells, and this immune memory can be maintained for several weeks to months. After re-stimulation by antigens, these long-lived memory NK cells rapidly proliferate and kill target cells (31–33).

Unlike CD8^+^ T cells, NK cell activation does not require antigen presentation but depends on the dynamic balance of activating and inhibitory signals from target cells, including cancer cells and infected cells (34). Cancer cells can escape the killing from cytotoxic T cells by downregulating the expression of MHC I molecules. However, this reduction of MHC I molecules on the surface of cancer cells triggers the activation of NK cells (35, 36). NK cells kill cancer cells mainly via four approaches. First, NK cells express FasL and TRAIL on the cell surface, which can bind to the corresponding receptors Fas and TRAILR on cancer cells, respectively. This binding triggers the activation of caspase-8 and, subsequently, caspase-3, leading to the apoptosis of target cells (37, 38). Second, NK cells release various cytotoxic granules such as perforin and granzyme B to induce lysis and apoptosis of tumor cells (37, 39–42). Third, the Fcγ receptor III or CD16 on NK cells can recognize the antibodies binding to cancer cells, which induces NK cells to produce more cytotoxic granules to eliminate cancer cells, an effect called antibody-dependent cell-mediated cytotoxicity (ADCC) (43–45). Fourth, NK cells can also secrete IFNγ to induce cancer cell death and regulate other cancer-fighting immune cells (46–48).

## Mechanisms of NK cell exhaustion in the tumor microenvironment

### Dysregulation of inhibitory and activating signaling

**Figure 1:**
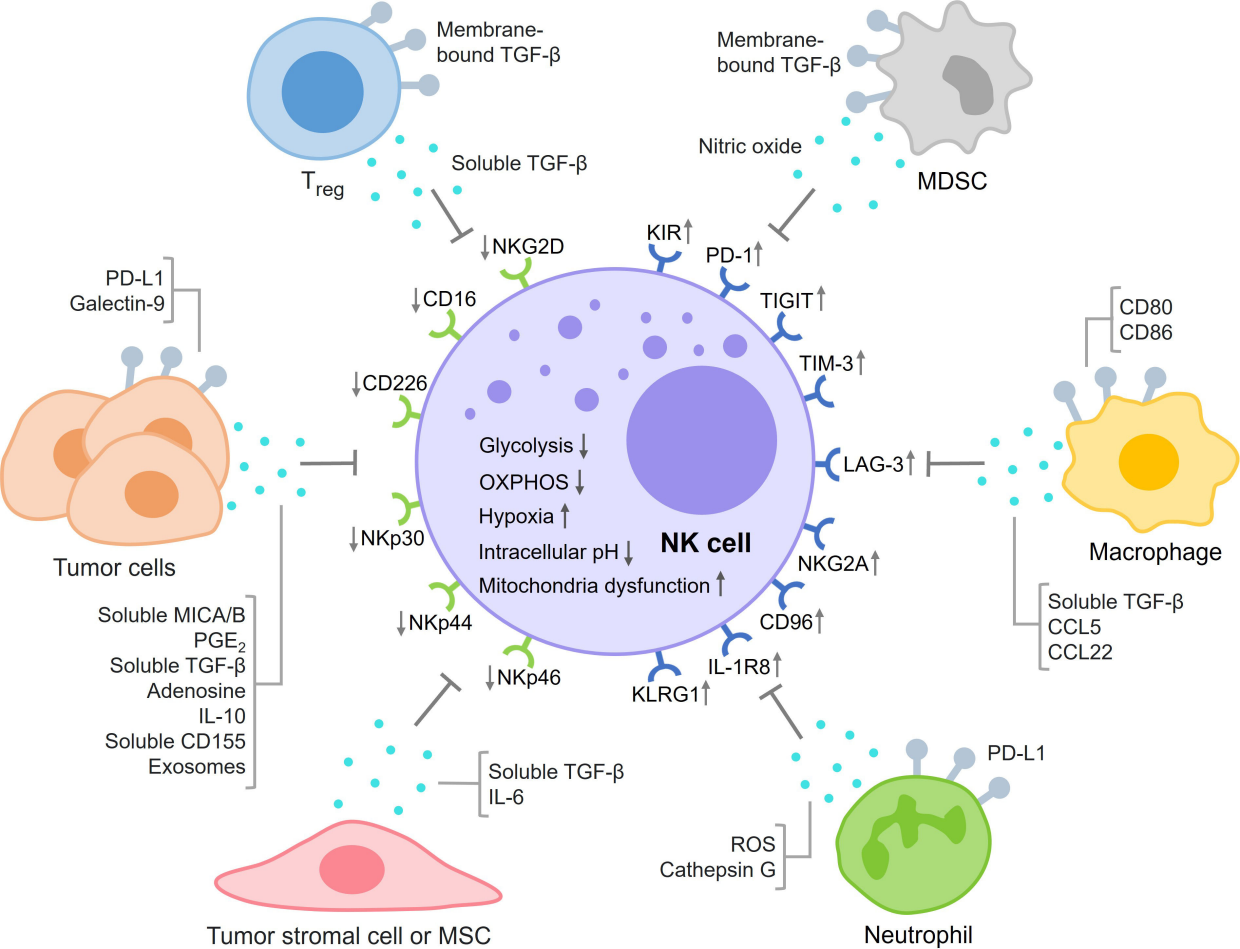
This figure summarizes the main mechanisms of NK cell exhaustion in the TME in four aspects: dysregulation of inhibitory and activating signaling, tumor cell-derived factors, immunosuppressive cells, and metabolic exhaustion.

The control of NK cell activation is a balance between signals from inhibitory receptors (such as KIR, PD-1, TIGIT, TIM-3, LAG-3, NKG2A, CD96, IL-1R8, and KLRG1) and activating receptors (such as NKG2D, CD16, CD226, NKp30, NKp44, and NKp46) (49–53). In the TME, inhibitory receptors on NK cells and their ligands on tumor cells are always upregulated, whereas activating receptors and ligands are downregulated (**Figure 1**). For example, in ovarian cancer patients, the percentage of PD-1^+^ NK cells in peripheral blood was much higher than in healthy individuals (54). When PD-L1 expressed on the surface of tumor cells bound to PD-1 on NK cells, an exhausted phenotype and even apoptotic cell death were induced in NK cells (55–57). TIGIT is a co-inhibitory receptor expressed on NK cells, and the major ligand for TIGIT is CD155 (58). NK cells in intratumoral regions showed significantly higher expression of TIGIT than the NK cells in peritumoral regions. The binding of CD155, expressed on tumor cells, to TIGIT on the surface of NK cells reduced the expression levels of IFNγ and the death ligand TRAIL by NK cells (59). TIM3 is a maturation marker for NK cells, and upregulation of TIM-3 was found in several cancers, including lung, colorectal, and gastric cancer (60–62). At least four ligands for TIM-3 have been found, including galectin-9, CD66a, phosphatidylserine, and HMGB1 (63). The interaction between galectin-9 and TIM-3 was thought to suppress NK cell-mediated cytotoxicity in tumor tissues (64). However, some other studies suggested that galectin-9 might enhance NK cell activity (65, 66). NKG2D is a potent activating receptor expressed on the surface of NK cells. In hepatocellular carcinoma patients, a much higher DNA methylation frequency in the NKG2D promoter region was detected (67). Downregulation of NKG2D on NK cells was found in various cancers such as glioma, leukemia, head and neck cancer, and cervical cancer and contributed to the decreased activity of NK cells (68–71).

### Tumor cell-derived factors

Tumor cells can secrete various factors that inhibit NK cell activity (**Figure 1**). The binding of MICA/B on tumor cells and NKG2D on NK cells activates NK cells. However, several studies showed that tumor cells also produced and shed soluble MICA/B proteins that could bind to NKG2D and inhibit NK cell activation and cytotoxicity by mechanisms that are still not fully elucidated (72–74). Tumor cells secreted PGE_2_ to inhibit the killing effects of NK cells by decreasing the levels of NK receptors such as NKp30, NKp44, and NKG2D (75). In addition, PGE_2_ also reduced the killing effects of NK cells by suppressing IFNγ production (76, 77). TGF-β is an important immunosuppressive factor in balancing the immune response. Not surprisingly, tumor cells can secrete TGF-β to inhibit NK cell activation and cytotoxicity (78, 79). Some studies revealed that extracellular adenosine levels were increased in the TME, which could inhibit degranulation and cytokine production by NK cells (80–82). IL-10 is an important immunoregulatory cytokine with both protumoral and antitumoral effects. In its immunosuppressive role, IL-10 suppresses the expression of NKG2D ligands and increases the expression of inhibitory ligands on tumor cells to inhibit the activation and killing effects of NK cells (83, 84). Some studies showed that IL-10 could also enhance the activities of NK cells (85, 86). CD155 is a ligand for both activating and inhibitory receptors expressed on NK cells. The soluble form of CD155 secreted by melanoma cells was found to inhibit NK cell degranulation and cytotoxicity (87). Tumor cell-derived extracellular vesicles or exosomes can both activate and inhibit NK cell function, depending on the properties of the exosomes. Several studies have shown that tumor cells produce exosomes containing NKG2D ligands and TGF-β, which can impair NK cell proliferation and cytotoxicity (88–90). Tumor cells can also generate exosomes containing noncoding RNAs such as miR-92b, SNHG10, and circUHRF1 to inhibit NK cell activity in the TME (91, 92).

### Immunosuppressive cells

In the TME, there are a number of cells in addition to tumor cells and NK cells (**Figure 1**) (93). CD4^+^CD25^+^ regulatory T cells (Tregs), as immunosuppressive cells, impaired the proliferation and IFNγ production of NK cells through TGF-β signaling (94, 95). Myeloid-derived suppressor cells (MDSCs) in the TME could also inhibit NK cell activity through membrane-bound TGF-β, which decreased the expression levels of NKG2D and IFNγ in NK cells (96). MDSCs increased the level of arginase-1, which reduced IFNγ production but did not affect granzyme B release or NK cell viability (97). In clinical patients with hepatocellular carcinoma, MDSCs mediated the suppression of NK cells through NKp30 (98). Nitric oxide produced by MDSCs inhibited NK cell cytotoxicity by impairing the ADCC response (99). Tumor-associated macrophages (TAMs) play an important role in tumor immune evasion. Several studies showed that TAMs induced NK cell dysfunction through secretion of TGF-β (100, 101). TAMs also inhibited NK cell activity by expressing CD80 and CD86, which bound to CTLA-4 on NK cells. In addition, immunosuppressive chemokine ligands such as CCL5 and CCL22 secreted by TAMs could recruit Tregs to the TME, further suppressing the killing effects of NK cells (102). There are sufficient numbers of neutrophils in the TME to interfere with NK cell activity. For example, neutrophils reduced the expression of CCR1 on NK cells, which could reduce the infiltration of NK cells into tumor tissues. The PD-L1 molecules on neutrophils bound to PD-1 receptors on the surface of NK cells, reducing the amount of IFNγ expressed by NK cells (103). Neutrophils could also release ROS and cathepsin G to inhibit NK cell cytotoxicity (104, 105). In addition to immune cells, tumor stromal cells have been suggested to inhibit the proliferation of NK cells and suppress the expression of NKp44 and NKp46 on NK cells (106). While mesenchymal stromal cells (MSCs) initially promoted NK cell function by releasing type I interferon, they later induced NK cell dysfunction by secreting TGF-β and IL-6, ultimately leading to NK cell senescence (107).

### Metabolism and exhaustion

Because of their unique metabolic properties, solid tumor cells generate various metabolic stresses such as nutrient depletion, low oxygen, low pH, and accumulation of waste products in the TME. These stresses not only affect the tumor cells but also severely impair the activity of NK cells in the TME (**Figure 1**) (108, 109). Adequate energy supply is important for proliferation, survival, and cytotoxicity of NK cells. Some studies suggested that steady-state NK cells preferred oxidative phosphorylation (OXPHOS) for ATP production, whereas activated NK cells relied more on glycolysis (109, 110). Disruption of either glycolysis or OXPHOS may impair NK cell function. Lung tumor cells secreted TGF-β in the TME, which upregulated the expression of fructose-1,6-bisphosphatase in NK cells, resulting in inhibition of glycolysis and decreased cytotoxicity and viability of NK cells (111). In melanoma, inhibition of Srebp activity led to a decrease in cytokine-enhanced glycolysis and OXPHOS in NK cells, which impaired the effector function of NK cells (112). Inhibition of glycolysis and OXPHOS could decrease the secretion of IFNγ by NK cells and attenuate their cytotoxicity to leukemia cells (113).

Hypoxia in the TME contributes to cancer cells escaping NK cells’ killing and developing into advanced tumors. Hypoxia inhibited the expression of heat shock protein 70 and MICA/B of tumor cells, which helped tumor cells escape recognition by NK cells (114). In multiple myeloma, hypoxia inhibited the expression of perforin and granzyme B by NK cells, impairing NK cell cytotoxicity (115). Hypoxia led to the upregulation of HIF-1ɑ and downregulation of NK cell activating receptors such as NKG2D and natural cytotoxicity receptors (116). In addition to NK cells, hypoxia-induced HIF-1ɑ could also increase the expression of PD-L1 on MDSCs, leading to the suppression of T cells (117). A liver cancer study showed that hypoxia induced activation of mTOR-Drp1 proteins in the tumor-infiltrating NK cells, leading to increased mitochondrial fragmentation in these NK cells. As a result, tumor-infiltrating NK cells exhibited lower cytotoxicity and were more prone to cell death compared with NK cells in tumor-adjacent tissues (118).

Most tumor cells rely on aerobic glycolysis for energy production, which is known as the “Warburg effect,” producing a large amount of lactate as a waste product (119). Several studies have found that lactate can lead to the acidification of the TME and impair NK cell activity. For example, melanoma cells highly expressed lactate dehydrogenase, an enzyme that catalyzes lactate synthesis from pyruvate, and the accumulation of lactate in melanomas decreased the number of NK cells and inhibited cytokine production by NK cells (120). To form liver metastases, colorectal cancer cells inhibited the function of liver-resident NK cells by secreting lactate, which lowered pH and damaged mitochondria inside NK cells (121).

Lipid metabolism is critical to the effector function of NK cells. A study showed that obesity-induced accumulation of lipids in NK cells decreased their anti-tumor activity by inhibiting cytotoxic machinery trafficking (122). In B-cell lymphomas, rich fatty acids in the TME impaired IFNγ secretion by NK cells (123). The formation of immunological synapses is critical for recognizing and killing cancer cells by NK cells. In liver cancer, inhibition of sphingomyelin biosynthesis in intratumoral NK cells severely dampened the membrane topology and synapse formation, which reduced NK cell cytotoxicity (124). Amino acid metabolism also regulates NK cell activity. Several works showed that tryptophan-derived kynurenine catalyzed by indoleamine 2,3-dioxygenase (IDO) inhibited NK cell proliferation and decreased activating receptors, including NKp46 and NKG2D, on the surface of NK cells (125, 126). IDO could also catalyze tryptophan to kynurenine in cancer cells, decreasing the expression of NKG2D ligands on the surface of cancer cells by ADAM10, and this reduced expression of NKG2D ligands inhibited degranulation and IFNγ release by NK cells (127).

## Therapeutic approaches to overcome NK cell exhaustion

Recombinant cytokine drugs are being developed to boost NK cell activation. IL-2 is a potent stimulator of NK cell and cytotoxic T cell survival and increases their killing activity. Several recombinant IL-2 drugs are currently under investigation or approved for the treatment of various cancers, such as lung, bladder, ovarian, and renal cell cancers (128–131). IL-12 is secreted mainly by antigen-presenting cells and enhances the proliferation, survival, and cytotoxicity of NK cells. Recombinant IL-12 is currently being tested in clinical trials for the treatment of head and neck cancer in combination with cetuximab, an EGFR inhibitor (132, 133). IL-15 has similar functions to IL-2 but does not have the activation-induced cell death effect (134). An IL-15 superagonist ALT-803 is being used in clinical trials to treat various types of cancers, including non-small-cell lung cancer, head and neck cancer, renal cell cancer, and melanoma (135, 136).

Immune checkpoint inhibitors have been used to block immunosuppressive signals on NK cells to enhance their anti-tumor activity. Blocking the PD-1/PD-L1 inhibitory axis has become a popular therapeutic strategy (137). PD-1/PD-L1 immune checkpoint inhibitors have been approved for the clinical treatment of various cancers (138, 139). Recently, a monoclonal antibody targeting LAG-3 has also been approved by the FDA for the treatment of melanoma (140). Dozens of monoclonal antibodies targeting other immune checkpoints, such as KIR, TIGIT, TIM-3, and NKG2A, are currently being developed and clinically tested (141–143). In addition to immune checkpoint inhibitors, agonistic antibodies are also being developed to activate costimulatory receptors on NK cells to enhance their anti-tumor activity. For example, monoclonal antibodies targeting 4-1BB are being tested in clinical trials for the treatment of lymphoma, melanoma, and non-small-cell lung cancer (144, 145).

Adoptive transfer of NK cells represents another strategy for cancer treatment. Therapeutic NK cells can be purified from peripheral blood or umbilical cord blood or derived from induced pluripotent stem cells. Some NK cell lines, such as NK-92, can also be used for transfusion (146, 147). Prior to transfusion, several cytokines such as IL-2, IL-12, IL-15, and IL-21 and feeder cells are used to promote NK cell activation and proliferation (148, 149). Genetically engineered NK cells such as chimeric antigen receptor (CAR)-NK cells and TCR-NK cells are generated to improve the killing ability and specificity of NK cells (150, 151). For example, anti-CD19 CAR-NK cells have been used in clinical trials to treat lymphoid tumors (152). Synthetic biology methods are also being investigated to help NK cells overcome immunosuppressive TME (153). The development of novel therapies based on NK cells and the combination of existing strategies will contribute greatly to cancer research and treatment.

## Author contributions

HJ: Writing – original draft. HY: Writing – original draft. HX: Writing – original draft. KL: Writing – review & editing.
